# A Non-Invasive Continuous Blood Pressure Estimation Approach Based on Machine Learning

**DOI:** 10.3390/s19112585

**Published:** 2019-06-06

**Authors:** Shuo Chen, Zhong Ji, Haiyan Wu, Yingchao Xu

**Affiliations:** 1 College of Bioengineering, Chongqing University, Chongqing 400044, China; 20161902046@cqu.edu.cn (S.C.); 201719021053@cqu.edu.cn (H.W.); 20161913065@cqu.edu.cn (Y.X.); 2 Chongqing Medical Electronics Engineering Technology Center, Chongqing 400044, China

**Keywords:** pulse waveform, pulse transit time (PTT), genetic algorithm (GA), machine learning, mean influence value (MIV)

## Abstract

Considering the existing issues of traditional blood pressure (BP) measurement methods and non-invasive continuous BP measurement techniques, this study aims to establish the systolic BP and diastolic BP estimation models based on machine learning using pulse transit time and characteristics of pulse waveform. In the process of model construction, the mean impact value method was introduced to investigate the impact of each feature on the models and the genetic algorithm was introduced to implement parameter optimization. The experimental results showed that the proposed models could effectively describe the nonlinear relationship between the features and BP and had higher accuracy than the traditional methods with the error of 3.27 ± 5.52 mmHg for systolic BP and 1.16 ± 1.97 mmHg for diastolic BP. Moreover, the estimation errors met the requirements of the Advancement of Medical Instrumentation and British Hypertension Society criteria. In conclusion, this study was helpful in promoting the practical application of methods for non-invasive continuous BP estimation models.

## 1. Introduction

According to the World Health Statistics 2018 published by the World Health Organization, the number of deaths caused by cardiovascular diseases (CVDs) accounted for 44% of deaths due to noncommunicable disease (NCD) in 2016 globally, which is as high as 17.90 million deaths [1]. Hypertension is one of the strongest risk factors for CVDs. About 50%–75% of strokes and 40%–50% of myocardial infarctions are associated with elevated blood pressure (BP) [2]. As an essential parameter monitoring the heart and vascular functions of the human body, BP is highly important for the prevention and diagnosis of CVDs.

However, BP may fluctuate and is closely correlated with target organ damages in hypertension [3]. Emotional fluctuations, strenuous exercise, and irrational use of medicines can cause changes in BP [4]. Traditional clinic-based measurements often fail to evaluate patients’ true BP objectively because of “white-coat hypertension” or “masked hypertension” [5]. Besides, the clinically commonly used oscillometric-based measurement technologies, such as mercury sphygmomanometer, electronic sphygmomanometer, and dynamic sphygmomanometer, can only intermittently measure BP and are not able to provide continuous long-term BP data [6]. However, the continuous BP measurement method, such as the direct BP measurement, overcomes the drawbacks of traditional measurement methods. It can complete BP measurement in each cardiac cycle and monitor BP changes more accurately. Therefore, it is recognized as the “gold standard” for BP monitoring internationally [7]. However, the direct BP measurement needs to be achieved by arterial catheterization, which makes it quite limited in clinical applications.

In recent years, non-invasive continuous BP monitoring technology has advanced greatly in every aspect. It ensures scientific, comprehensive, and accurate BP information, comes with good repeatability, and is widely used in intensive care, health monitoring, and medical research; hence, it is remarkably valuable in application and research. The commonly used methods include volume-clamp method, arterial tonometry method, ultrasonic method, and pulse transit time (PTT) method, each of which has its own advantages and disadvantages and scope of application. The volume-clamp method is a relatively mature continuous BP measurement method on the market [8], but long-term measurement may cause venous congestion and discomfort in patients, affecting the measurement accuracy. The arterial tonometry method has a good accuracy [9], but it is sensitive to the sensor position that makes it difficult for continuous long-term BP measurement. The ultrasonic method has a strong anti-interference ability [10], but the complexity and high cost of the instrument are the primary factors hindering its development.

Hence, the PTT method has been favored by researchers because it is cuffless, convenient, and comfortable, and only needs electrocardiogram (ECG) electrodes and pulse sensors. Payne et al. explored the relationship of PTT with systolic BP (SBP) and diastolic BP (DBP) by altering the BP of the patients with medicines [11]. Their experiment revealed a good linear relationship of PTT with SBP, but not much with DBP. Dingli proposed a linear model for SBP estimation based on PTT, and simultaneously, constructed a DBP prediction model by integrating the obtained SBP values with the individualized parameters related to peripheral resistance and arterial compliance [12]. Based on PTT, Xuejun et al. selected a number of BP-related characteristic parameters of pulse waveform, such as the K value and pulse rate, to establish characteristic equations for different patients to estimate SBP by stepwise regression, but they did not estimate DBP [13]. Ding et al. introduced a photoplethysmogram (PPG) intensity ratio to estimate variations in arterial diameter, which could serve as a key indicator of DBP estimation [14]. In addition, numerous scholars introduced various characteristic parameters to improve the model accuracy of BP estimation, unfortunately with limited effects [15,16,17]. In summary, the aforementioned PTT and characteristic parameters of pulse waveform correlated with SBP and DBP in some way, but highly depended on the physiological characteristics of the patients and usually required individual calibration during measurements. These correlations could not be easily described with linear models and were poor in accuracy and robustness too.

Nowadays, researchers have attempted to build more complicated models for BP estimation based on big data to describe the correlation of PTT and characteristic parameters of pulse waveform with BP. Peng et al. proposed a wavelet neural network model trying to understand the relationship between pulse wave and arterial blood pressure (ABP), and then extracted SBP and DBP from reconstructed arterial BP waveform [18]. The results were in line with the Association for the Advancement of Medical Instrumentation (AAMI) criteria, but the method had high computational complexity and significant data redundancy. He et al. used the random forest algorithm to construct a BP estimation model, which was simple and effective, but the model performance was severely diminished for patients with high BP fluctuations [19]. Zhang et al. applied the gradient boosting decision tree to predicting BP rates, which had better performance in calculating the mean absolute error evaluation index than methods, such as the least squares method, ridge regression, lasso regression, etc. [20]. Kachuee et al. compared the performance of several different machine learning algorithms in BP estimation [21]. Additionally, some learning algorithms such as deep neural networks and gaussian mixture regression were applied to the oscillometric BP estimation [22,23]. In addition, some related studies accomplished good outcomes too [24,25].

This study aimed to construct more accurate continuous BP estimation models using the machine learning method with rich feature information related to PPG waveform and BP based on the accurately extracted PTT information. In this study, a total of 14 BP-related features, including PTT, were extracted from biosignals, and the correlations between the features and the initial BP estimation models were analyzed. Then, the BP estimation models were built by machine learning based on the features of dimensionality reduction, and the model parameters were optimized by optimization algorithms. In the end, whether the present models have higher estimation accuracies than the traditional multiple linear regression models and the PTT-based BP estimation models was explored, and conclusions were reached according to AAMI and the British Hypertension Society (BHS) criteria.

## 2. Materials and Methods

### 2.1. Data Collection

**Figure 1:**
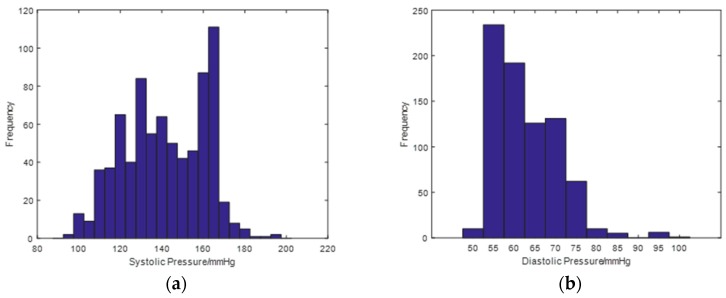
(**a**) Distribution of systolic blood pressure. (**b**) Distribution of diastolic blood pressure.

The data in this study were retrieved from the MIMIC-III Waveform Database, a multiparameter critical care database open to the public at the Massachusetts Institute of Technology (MA, USA) [26]. The MIMIC-III Waveform Database Matched Subset contained 22,317 waveform records and 22,247 numeric records from patients; some of the waveform records provided biosignals, including ECG, PPG, and ABP signals, captured simultaneously by monitoring beds in the intensive care unit. The database not only ensured data diversity but also offered ABP signals as a standard comparison, providing a solid data foundation for the construction of BP estimation models. In this study, a total of 772 sets of waveform data were acquired online using the WFDB Toolbox in which the function RDSAMP allowed users to load PhysioNet waveform data into MATLAB’s workspace [27]. All experiments in this study were based on the MATLAB platform. The distribution of BP in the experimental dataset is shown in Figure 1. The plots demonstrated that the data were widely distributed, covering from low BP to high BP values, and could be used to effectively evaluate the predictive power of the models.

### 2.2. Preprocessing and Feature Extraction

The ECG and PPG signals provided by the MIMIC-III Waveform Database were weak electrical signals, susceptible to myoelectric interference and baseline drift during signal collection, and affected the accuracy of signal feature extraction. Therefore, to maximize the useful information of the original signals, after removing the data segments with irregularities and missing waveforms in the database, the wavelet threshold denoising method and the cubic spline interpolation method were used, respectively, to denoise the ECG and PPG signals [28].

**Figure 2:**
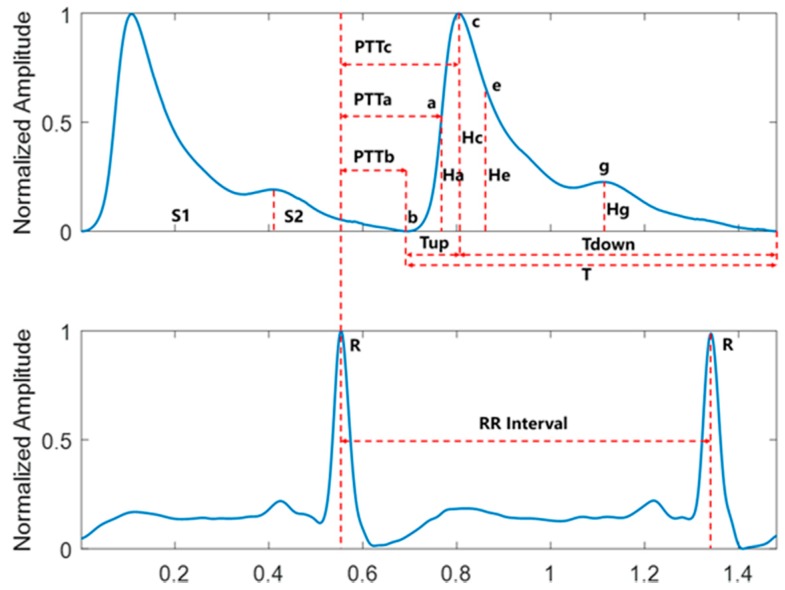
Schematic diagram of features.

To construct a dataset for the BP estimation models, it was necessary to accurately extract the features of the original signals and select effective features, improving the generalization and reducing overfitting. In this study, 14 BP-related features, including PTT, heart rate (HR), and characteristics of pulse waveform, were selected, as shown in Figure 2. The detailed relationships and definitions are as follows:

#### 2.2.1. PTTx Features

In 1979, Hughes proposed a theoretical model describing the relationship between BP and Young’s modulus of elasticity of aorta, and defined as follows [29]:

(1) $$E=E_{0}e^{\gamma P},$$

where *E_0_* is the Young’s modulus for zero pressure, *γ* is the vessel characteristic parameter, and *P* is the BP.

In arteries, the relationship between the elastic modulus of the arterial wall and the pulse wave velocity (PWV) can be expressed by the Moens–Korteweg equation:

(2) $$PWV=\frac{D}{PTT}=K\sqrt{\frac{tE}{\rho d}}$$

where *D* is the pulse transit distance, *K* is the Moens constant, *t* is the arterial wall thickness, *d* is the inner diameter of arteries in equilibrium, and *ρ* is the blood density.

Equations (1) and (2) together demonstrated a close correlation between PTT and BP. In this study, the PTT features were obtained by calculating the time from the R-peak of the ECG signal to the corresponding pulse feature point in each cardiac cycle. The following three PTT features were selected for this: the time from the R-peak to the pulse start point b (PTTb), the time from the R-peak to the point with maximum slope a (PTTa), and the time from the R-peak to the pulse peak point c (PTTc).

#### 2.2.2. *K* Value

The *K* value is closely related to the peripheral resistance and the hardening degree of the arterial wall, and is one of the important physiological indicators for clinical research of CVDs [30]. The *K* value is dimensionless, related only to the area of the pulse wave, and defined as follows:

(3) $$K=\frac{PPG_{m}-PPG\left(t_{b}\right)}{PPG\left(t_{c}\right)-PPG\left(t_{b}\right)},$$

where $PPG_{m}=\frac{1}{T}\int PPG\left(t\right)dt$.

#### 2.2.3. HR

If HR is accelerated while holding the cardiac output and peripheral resistance constant, due to the shortened diastole, the blood flows to the peripheral blood vessels is reduced while the blood in the aorta is increased, resulting in increased DBP and SBP, and vice versa. The HR in this study was calculated using the R–R interval obtained by calibrating the R-peak of the ECG signal.

#### 2.2.4. Characteristics of Pulse Waveform

The characteristics of pulse waveforms selected in this study included relative time for rising *Tupr* = *Tup/T*, relative time for falling *Tdownr* = *Tdown/T*, main wave rising slope *Cslope* = *Hc/Tup*, relative height of the maximum slope point *Har* = *Ha/Hc*, relative height of the minimum slope point *Her* = *He/Hc*, relative height of the dicrotic wave peak point *Hgr* = *Hg/Hc*, relative area of systole *S1*, relative area of diastole *S2*, and area ratio of systole to diastole *S1*/*S2*.

### 2.3. Normalization and Dimensionality Reduction of Features

For the preprocessed data, 80% of the data was randomly selected as the training set, and the remaining 20% was used as the test set. In machine learning, different feature vectors represent different evaluation indicators. These feature vectors usually have different dimensions; therefore, the data need to be normalized to improve the comparability among features. At the same time, normalization can reduce the adverse effects caused by outliers and speed up the gradient descent to find the optimal solution. In this study, the min–max normalization method was used to linearly transform the data to the range of [0, 1]. The mapping function is as follows:

(4) $$x^{\prime }=\frac{x-min\left(x\right)\;}{max\left(x\right)-min\left(x\right)\;}.$$

In this study, the training set and the test set were normalized altogether to effectively solve the inaccurate prediction problem due to the broad range of the training set and the narrow range of the test set.

To verify the validity of the features and remove redundant features, this study used the mean impact value (MIV) method to evaluate the importance of each feature to the BP estimation models and remove the features with small MIVs to achieve dimensionality reduction [31]. MIV could improve the efficiency of feature extraction while simplifying the model structure and upgrading the model performance. The sign of MIV represented the direction of correlation, and the absolute value represented the degree of the impact. In this study, two different feature combinations were selected as input to the SBP and DBP models, respectively. The detailed calculation process is as follows:

(1) After finishing the model training, two new training sets X1 and X2 were generated by transforming each input variable value of a feature in the training set X by ±10%. 

(2) The X1 and X2 were simulated to obtain the results P1 and P2, and the values of P1–P2 were calculated to get the impact values (IV) of the feature on the model output. The MIV was calculated by averaging the IVs by the number of observations. 

(3) The MIVs of all features were calculated and then sorted according to their absolute values. Then, the relative contribution ratio of each feature to the output was calculated, according to the following equation:

(5) $$I_{i}=\frac{\left|MIV_{i}\right|}{\sum_{i=1}^{k}\left|MIV_{i}\right|}.$$

### 2.4. Modeling Methods

Various factors influence the BP, and the relationships between features and BP are complicated and lack clear mechanisms. To adapt to the nonlinearity of the dataset and overcome the shortcomings of traditional fitting methods, the support vector regression (SVR) was used to construct the SBP and DBP estimation models. Further, the proposed models were compared with the traditional models using the Multivariate Linear Regression (MLR) models and the PTTc-based SVR models.

#### 2.4.1. SVR

SVR had many unique advantages in solving small sample and nonlinear regression problems. In the SVR, the input sample *x* was mapped into a high-dimensional feature space by the nonlinear mapping *Φ*(*x*), and then a linear model was built in this feature space to estimate the regression function. The equation used was as follows:

(6) f(x,ω)=ω·Φ(x)+b,

where ***ω*** is the weight vector and *b* is the threshold. In this study, for a given training set, the *ε*-insensitive loss function was used, and the corresponding SVR was called *ε*-SVR. Thus, the following constrained optimization problems needed to be solved:

(7) $$min\;\frac{1}{2}{\left\|\omega \right\|}^{2}+c\sum_{i=1}^{l}\;\left(\xi_{i}+\xi_{i}^{*}\right),\;\mathrm{such}\;\mathrm{that}\;\{\begin{array}{c} y_{i}-\left(\omega^{T}x_{i}+b\right)<\varepsilon +\xi_{i}\\ \left(\omega^{T}x_{i}+b\right)-y_{i}<\varepsilon +\xi_{i}^{*}\\ \xi_{i},\;\xi_{i}^{*}\geq 0 \end{array},$$

where *c* is a penalty factor, and $\xi_{i}$ and $\xi_{i}^{*}$ are different relaxation factors. For easier computation, the Lagrange multipliers were introduced to transform the aforementioned constrained optimization problems into a dual problem. The solution of Equation (6) was as follows:

(8) $$f\left(x\right)=\overset{l}{\underset{i}{\sum }}\left(-\alpha_{i}+\alpha_{i}^{*}\right)K\left(x_{i},x\right)+b,$$

where, $\alpha_{i}$ and $\alpha_{i}^{*}$ are Lagrange multipliers corresponding to support vectors (SVs), l is the number of SVs, and $K\left(x_{i},\;x\right)$ is a kernel function. However, in SVR, different kernel functions had great impacts on the fitting results. The preferred kernel function in this study was the radial basis function (RBF), which was as follows:

(9) $$K\left(x_{i},x\right)=exp\;\left(-\gamma {\left\|x-x_{i}\right\|}^{2}\right),$$

where γ is the kernel parameter. Compared with the linear kernel, the RBF kernel projected the dataset into a higher-dimensional space nonlinearly and “nonlinearized” the original linear algorithm, making it possible to effectively deal with the nonlinear relationship between features and BP. Compared with the polynomial kernel, it had fewer tuning parameters and reduced the complexity in model selection. 

Equations (7) and (9) indicate that selecting an appropriate penalty factor c, kernel parameter γ, and loss function ε could effectively improve the expansion of the SVR model. However, no uniform guidelines were present for parameter selection. How to quickly and effectively choose parameters was the key to the predictive power of the model. In this study, the LIBSVM toolbox was used to perform SVR model construction [32].

#### 2.4.2. MLR

MLR has been widely applied as a method to analyze the correlation, correlation direction, and strength between multiple independent variables and the dependent variable. In this study, an MLR model was built for the comparative purpose, and the model parameters were estimated using the least squares method. The model was expressed as follows:

(10) $$P=\beta_{0}+\beta_{1}x_{1}+\beta_{2}x_{2}+\ldots +\beta_{k}x_{k},$$

where P is BP, $\beta_{1},\beta_{2},\ldots ,\beta_{k}$ are model parameters, and $x_{1},x_{2},\ldots ,x_{k}$ are input features.

### 2.5. Parameter Optimization

For the aforementioned SVR model parameters, the traditional method allowed *c*, *γ*, and *ε* to take values within a certain range. Then, according to a set of selected parameters, the training set was used as the original dataset to calculate the model accuracy, and the set of parameters giving the highest accuracy was taken as the optimal parameters. Besides being time-consuming and laborious, this method tended to fall into a local optimal solution, which was not good for parameter optimization in a wide range. Therefore, the genetic algorithm (GA) was used in this study for parameter optimization.

**Figure 3:**
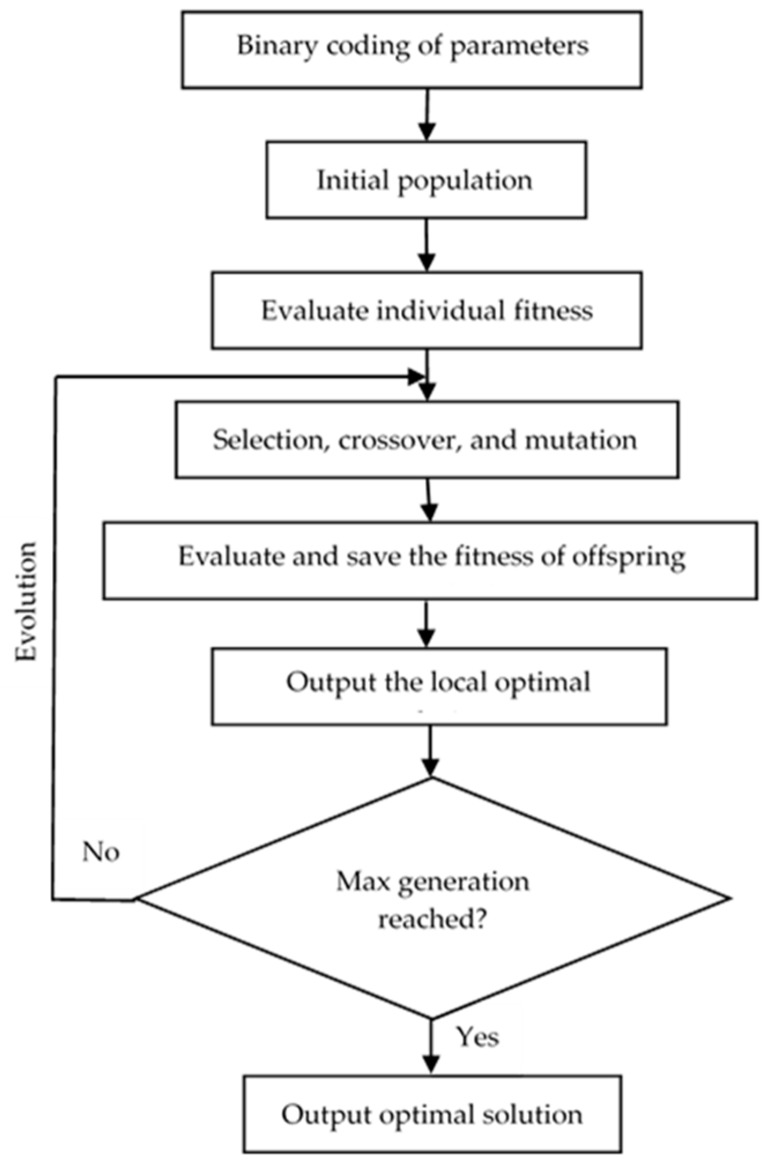
Flowchart of the genetic algorithm optimization.

GA is a computational model that simulates the evolutionary process according to the natural selection of Darwin’s theory of evolution and the genetic mechanisms, which includes a method of searching for optimal solutions by simulating the natural process of evolution. Adopting the probabilistic optimization method, GA could automatically acquire and direct the optimized searching space with no definite rule and adjust the searching direction adaptively. It had a superior global optimization capability. The algorithm flowchart is shown in Figure 3. The detailed steps were as follows:

(1) Binary coding. Binary coding of *c*, *γ*, and *ε* was performed to make a binary string, which served as a corresponding individual solution in the solution space, that is, the parameter ranges. Individuals made up a population.

(2) Initial population. A population was randomly generated and input into the fitness function to calculate and evaluate the fitness score of each individual.

(3) Selection, crossover, and mutation. For the initial population, the selection was performed according to the fitness scores, crossing over according to the crossover probability, and mutating according to the mutation probability to generate an offspring population.

(4) Evaluation and saving of the fitness of offspring individual. The fitness score of each individual was evaluated in the offspring population, and the local optimal solution was output.

(5) Output optimal solution. Once the max generation of the genetic operation was reached, the decoded optimal parameters were output. 

The parameters for GA optimization were set as follows: [0, 100] for *c*, [0, 1000] for *γ*, and [0.01, 1] for *ε*; 20 for initial population, 0.7 for crossover probability, 0.01 for mutation probability, and 100 for max generation.

### 2.6. Models Validation and Evaluation

In this study, the average mean squared error (average MSE) obtained from the fivefold cross-validation (5-CV) was used to measure the model accuracy.

(11) $$Average\;MSE=\frac{1}{5}\sum_{j=1}^{5}MSE_{j},\;MSE=\frac{1}{n}\sum_{i=1}^{n}{\left(y_{i}-\hat{y_{i}}\right)}^{2}.$$

This meant that the training set was randomly divided into five groups; each group was used as the test set, and the remaining four groups were trained to get the SVR model. The MSE values from the five validations were averaged to get the average MSE. 

The mean absolute deviation (MAD) and the standard deviation (STD) were treated as indicators for evaluating the predictive performance of models.

(12) $$\mathrm{MAD}=\frac{1}{n}\overset{n}{\underset{i=1}{\sum }}\left|y_{i}-\hat{y_{i}}\right|,$$

(13) $$\mathrm{STD}=\sqrt{\frac{1}{n-1}\overset{n}{\underset{i=1}{\sum }}{\left(y_{i}-\hat{y_{i}}-\bar{y_{i}-\hat{y_{i}}}\right)}^{2}},$$

where $y_{i}$ is the actual value of BP and $\hat{y_{i}}$ is the predicted value of BP. 

## 3. Results

### 3.1. Preprocessing Results

**Figure 4:**
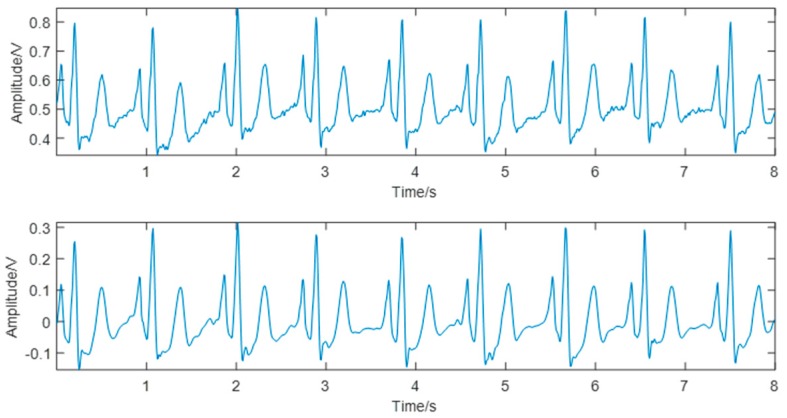
Comparison of the electrocardiogram (ECG) signal before (**top**) and after (**bottom**) denoising.

The ECG signals collected contained two major types of noise: myoelectric interference and baseline drift. In this study, the Symlet 8 (sym8) wavelet was used to decompose the ECG waveform into eight scales. The approximate component of scale 8 was directly zeroed to remove the baseline drift partially, and the detail component of scale 1 was directly zeroed to remove part of the myoelectric interference. The other components were processed by combining the “sqtwolog” fixed threshold and the soft threshold function to effectively eliminate the noise and retain the main components of the waveform. The denoising results are shown in Figure 4.

**Figure 5:**
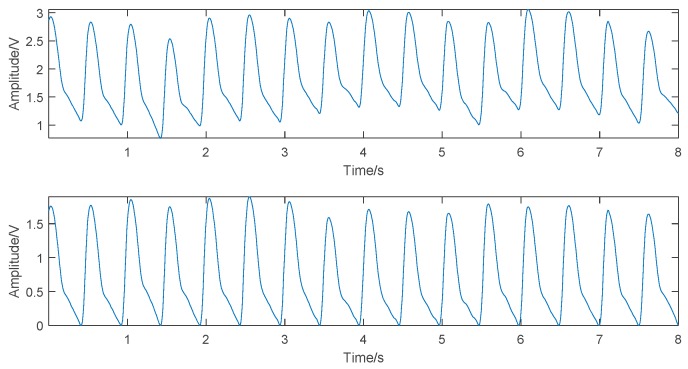
Comparison of the photoplethysmogram (PPG) signal before (**top**) and after (**bottom**) denoising.

For a major baseline drift in PPG, according to the pulse valley positions in the original signal, this study first used the cubic spline interpolation method to fit the drifting baseline and then subtracted the fitted curve from the original signal to get the baseline drift-removed PPG. The PPG denoising results are shown in Figure 5.

### 3.2. Model Construction

#### 3.2.1. GA-SVR BP Models

In this study, first the training set was used to construct the initial SVR models, with default values for all parameters. However, the experiments showed poor accuracies of the initial SVR models. Taking the SBP model as an example, the average MSE obtained from 5-CV was 337.37, clearly showing underlearning, indicating that the default parameters failed to produce an effective model. Therefore, GA was used for parameter optimization to boost model accuracy and avoid overlearning or underlearning. At the same time, it contributed to the faithful description of the feature impact on the model during the later dimensionality reduction.

**Figure 6:**
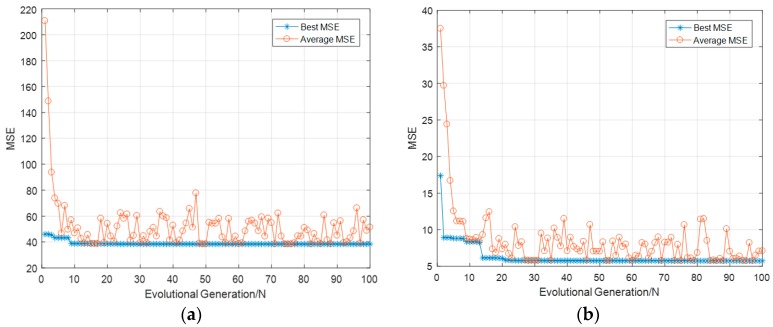
Genetic algorithm (GA) optimization process for initial models, the mean squared error (MSE) served as the fitness value of individuals in each generation. (**a**) Systolic blood pressure (SBP) model. (**b**) Diastolic blood pressure (DBP) model.

During optimization, the average MSE calculated by 5-CV also served as the fitness value of individuals in each generation for fitness evaluation, as shown in Figure 6. The average MSE and best MSE curves for all generations during evolution were plotted. From the best MSE curve, it was seen that the predictive ability of the SBP estimation model stabilized after 10 generations, while that of the DBP model stabilized after 20 generations.

The parameters obtained by GA optimization, the default parameters, and the model accuracies before and after optimization are shown in Table 1. It was seen that the accuracy enhanced by GA optimization was significant. The best MSE of the SBP estimation was improved to 38.33, and the best MSE of the DBP estimation was improved to 5.73. Therefore, the GA optimization was necessary before the features were subjected to MIV dimension reduction.

#### 3.2.2. GA-MIV-SVR BP Models

The constructed GA-SVR BP model already achieved a certain accuracy. It described the actual impact of features on the model in the study of the feature MIVs. The calculated MIV, relative contribution ratio, and cumulative contribution ratio of each feature with respect to the SBP and DBP models are listed in Table 2 and Table 3. It was seen that the MIVs of those two models ranked differently. This demonstrated that the correlations of each feature with respect to SBP and DBP were different, consistent with the general physiological rules.

**Figure 7:**
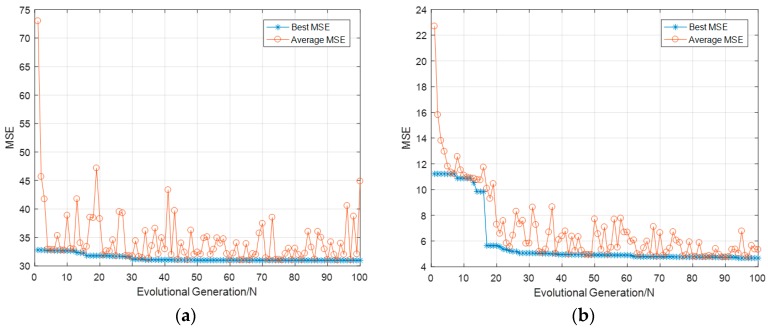
GA optimization process after dimensionality reduction, the MSE served as the fitness value of individuals in each generation. (**a**) SBP model. (**b**) DBP model.

In this study, the features contributing to 90% of cumulative contribution ratio were retained, and those with small MIVs were removed. It resulted in the first eight features in Table 2 being kept for the SBP estimation model and the first nine in Table 3 for the DBP model. The reduction in features could effectively increase the efficiency of feature extraction. Meanwhile, the feature reduction resulted in a new dataset, and the models based on the previous dataset needed to be modified accordingly. Therefore, to find the optimal parameters adapted to the new dataset and investigate whether the model accuracy was enhanced after dimensionality reduction, another round of GA optimization was needed for establishing the final BP estimation models. Similarly, the GA optimization process after dimensionality reduction is shown in Figure 7. The model accuracy comparison before and after the MIV process is shown in Table 4. It was seen that the best MSEs were further reduced. Although the accuracy improvements in the present round using the GA-MIV-SVR models were smaller than those in the last round using the GA-SVR models, it still proved that the MIV feature dimensionality reduction was effective, providing strong evidence for the correlation analysis between features and models.

### 3.3. Model Robustness and Comparison

The robustness of the constructed BP estimation models was verified using the test set with 20% data. To evaluate the proposed models in an objective manner, the MLR models and the PTTc-based SVR models were constructed from the same dataset and compared with the corresponding proposed models.

**Figure 8:**
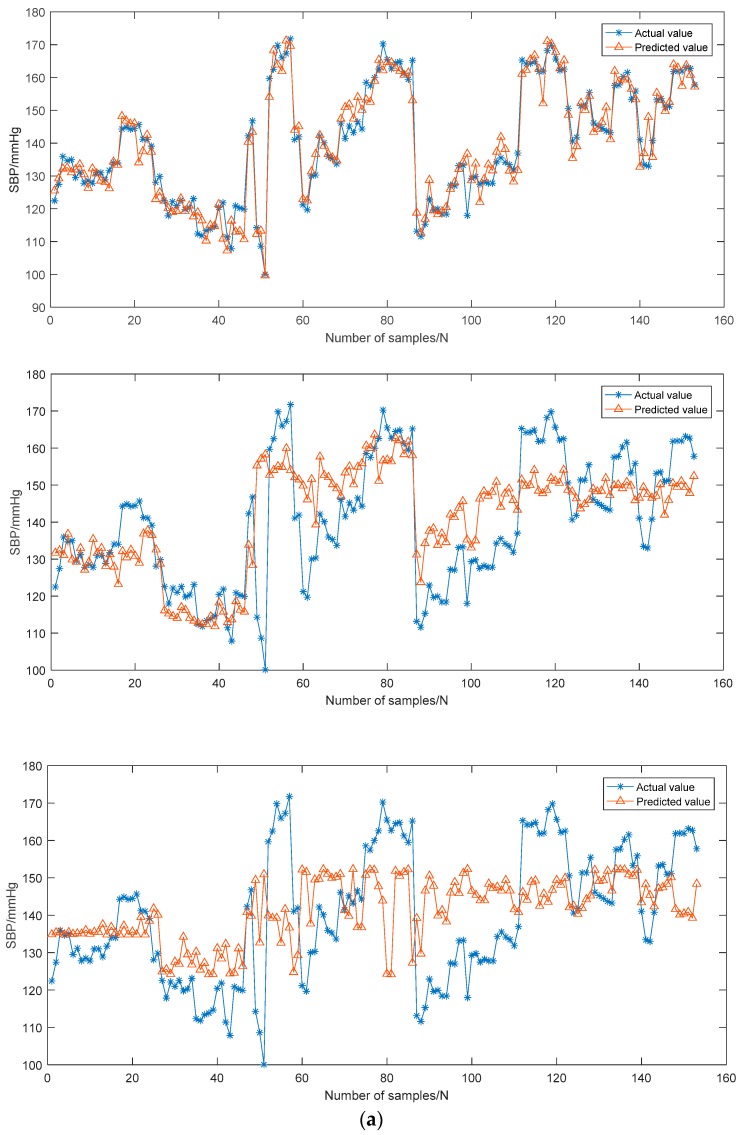

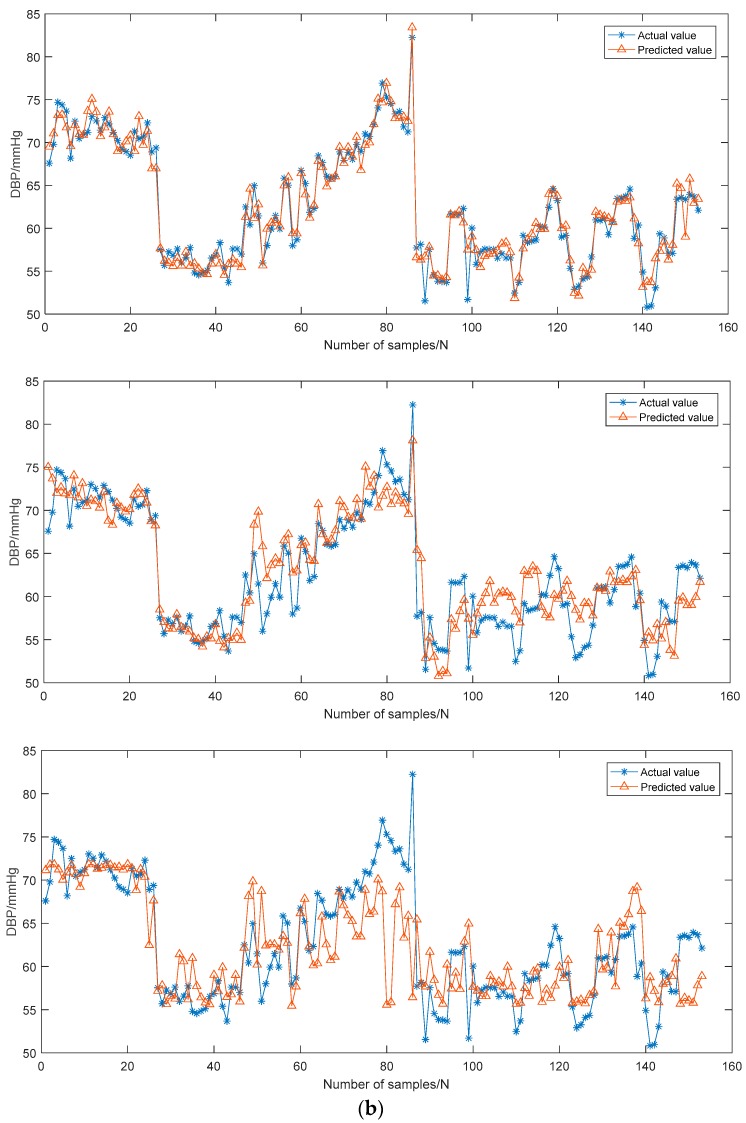
Comparison of predicted values and actual values. (**a**) SBP models. (**b**) DBP models. For both (a) and (b), from top to bottom, using GA-MIV-SVR, traditional multivariate linear regression (MLR), and pulse transit time to point c (PTTc)-SVR models.

The robustness comparison among the GA-MIV-SVR BP models and the traditional MLR BP models and the PTTc-based SVR BP models is shown in Figure 8 and Table 5. It was seen that the final constructed SBP and DBP estimation models by GA-MIV-SVR in this study generated predicted values closest to the actual values, with the highest prediction accuracies among the three methods. The SBP error was 3.27 ± 5.52 mmHg, and the DBP error was 1.16 ± 1.97 mmHg, fully satisfying the AAMI criteria with mean error ≤5 mmHg and STD ≤8 mmHg. Although the DBP estimation errors by the other two methods met the criteria, the SBP errors failed. In addition, the three prediction accuracies were evaluated according to the BHS grading system, as shown in Table 6. The results demonstrated that the cumulative errors of the SBP model constructed by the GA-MIV-SVR method were 77.8% (≤5 mmHg), 96.7% (≤10 mmHg), and 99.3% (≤15 mmHg), respectively, and of the DBP models were 98.7% (≤5 mmHg), 100% (≤10 mmHg), and 100% (≤15 mmHg), respectively, both met the grade A criteria of the BHS system.

**Figure 9:**
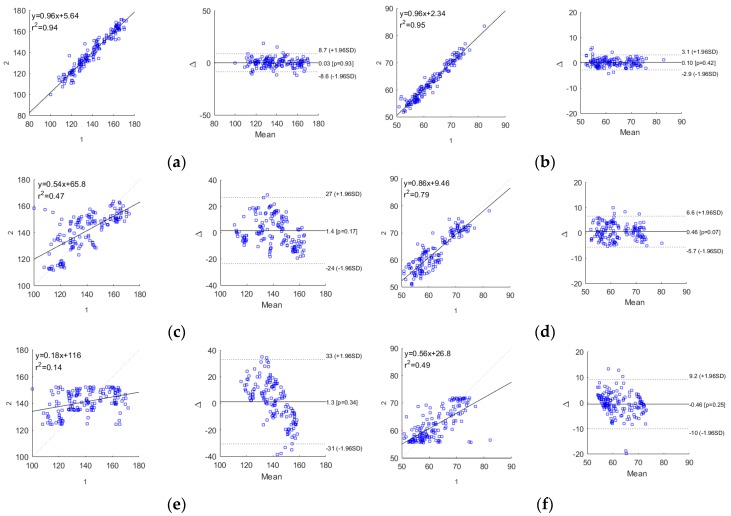
Correlation and Bland–Altman analysis. (**a**) GA-MIV-SVR SBP model. (**b**) GA-MIV-SVR DBP model. (**c**) Traditional MLR SBP model. (**d**) Traditional MLR DBP model. (**e**) PTTc-SVR SBP model. (**f**) PTTc-SVR DBP model.

To better analyze the correlations between the predicted and the actual values of SBP and DBP models, the Pearson correlation coefficient ($r^{2}$) and the Bland–Altman plots were both introduced. It was generally considered that 0.2 < $r^{2}$ < 0.4 indicated a weak correlation, 0.4 < $r^{2}$ < 0.6 a medium correlation, 0.6 < $r^{2}$ < 0.8 a strong correlation, and 0.8 < $r^{2}$ < 1.0 an extremely strong correlation. Figure 9 exhibits the Pearson correlation coefficient distributions and the Bland–Altman plots for the predicted and actual values of the SBP and DBP models by all three methods. It was shown that the models by GA-MIV-SVR showed extremely strong correlations between the predicted and the actual values, superior to the other two methods, indicating a highly linear relationship between the predicted values and the actual values. Besides, the Bland–Altman plots exhibited that for the SBP and DBP models by GA-MIV-SVR, the mean difference was 0.03 and 0.10 (close to 0) and 95.4% and 96.7% predicted values fell within the 95% limits of agreement, respectively, the [$\bar{d}-1.96SD,\;\bar{d}+1.96SD$]. This demonstrated that the predicted and the actual values were highly consistent, and the models proposed in this study could be used for non-invasive continuous BP estimation clinically.

## 4. Discussion

In this study, non-invasive continuous BP estimation models were proposed based on machine learning. Different from the traditional PTT-based BP estimation models, more BP-related features were introduced to model construction via information fusion. The proposed method for model construction effectively boosted the accuracy of BP estimation and enhanced the predictive performance and generalization ability of the BP estimation models.

### 4.1. Basis for Feature Selection and Dimensionality Reduction

The formation of BP in the human body is directly related to factors such as cardiac output, peripheral resistance, and degree of arteriosclerosis. In terms of feature selection, in addition to the features already proved to be related to the factors, such as PTT and *K* values, the features proposed in this study were more or less associated with these factors too. Although no definitive experiments proved the physiological mechanisms of correlation, some inferences made were based on physiological rules and related studies. For example, *Tupr* corresponded to the rapid ejection phase of the left ventricle; therefore, presumably it was related to cardiac output. Keeping cardiac output and peripheral resistance constant, changes in HR caused variations in BP. *Cslope* might be associated with blood viscosity [33]. *Her* and *Hgr* could tell something about aortic compliance [29]. *S1* and *S2* could also describe some characteristics of vascular elasticity and blood viscosity [34,35].

Based on the aforementioned data, this study attempted to describe the nonlinear relationship between features and BP by exploring the impact of each feature on the models. Compared with the use of the traditional Pearson correlation coefficient to describe the linear relationship, the MIV method was more rigorous and consistent with physiological rules. For example, the MIV of the *K* value was the largest in the SBP estimation model, while clinically the *K* value served as the key feature of the SBP estimation. The MIV of HR was the biggest in the DBP model, exhibiting the impact of HR on DBP. Of course, speculations proved to be unreasonable. For instance, the MIVs of *Tupr* ranked low in both SBP and DBP estimation models; hence, it was discarded in the subsequent study. In the absence of definite physiological mechanisms, the MIV method used in this study provided strong evidence and sufficient rationale for feature selection and dimensionality reduction. The MIV method not only described the nonlinear relationship between features and BP but also removed the redundant features, reducing the risk of insufficient generalization capabilities due to the inclusion of unrelated features.

The feature selection in this study still had some limitations. In the data collection stage, bounded by the database, the individual’s personal information could not be retrieved as features. It was expected that the addition of some personal characteristics, such as height, weight, age, and gender, might further strengthen the predictive ability of the models. In a previous study [36], the body mass index, waist circumference, hip circumference, and waist-to-hip ratio were introduced to predict the increase in BP. In a previous study [37], the BP neural network was constructed based on the height, weight, age, gender, and other characteristics for BP estimation. Furthermore, the introduction of personal features might possibly eliminate the cumbersome calibration process.

### 4.2. Model Methods and Limitations

Compared with the traditional PTT-based linear models [12], this study introduced some reasonable PPG waveform features for model construction and effectively improved the accuracy of SBP and DBP estimations. Compared with the MLR models [13,38], the models established in this study based on machine learning demonstrated a more complicated nonlinear relationship between the waveform characteristics and BP and greatly enhanced the predictive ability and robustness of the models.

From the perspective of the proposed method, this study adjusted the model parameters by GA optimization to achieve a better performing model. Compared with the traditional manual adjustment of parameters or directly using the default values, the proposed models had minimum underlearning or overlearning, greatly improving the optimization efficiency and facilitating model calibration. From the application scenario, the proposed models were satisfactorily applied in the continuous non-invasive monitoring of BP clinically. Getting rid of the constraints of the traditional cuff sphygmomanometer, these models helped doctors to gather more information about the changes in BP of the patients and get early warnings of diseases, leading to better management of BP.

However, the model method of this study had some limitations. First, as mentioned earlier, no personal information was introduced as features, and the study could not eliminate the influence of individual differences on the models. Second, no accuracy verification of long-term monitoring was conducted. It is yet impossible to know whether the accuracy of BP estimations will decrease when the models are monitored for a long time, for example, 1 week, 1 month, or 6 months. Last but not least, the data size in this study was not big enough. The diversity and size of data need to be further expanded. Additionally, the generalization ability of the models was affected to a certain degree.

## 5. Conclusions

This study integrated the characteristics of pulse waveforms and proposed a set of more effective BP estimation models using the GA-MIV-SVR method. The feature selection relied heavily on the characteristics describing the BP formation. The MIV measured the impact of features on the models to reduce the feature redundancy. In addition, the optimization method was introduced to improve the efficiency of parameter optimization; it effectively boosted the accuracies of SBP and DBP estimations by reducing underlearning or overlearning. This study was helpful for the wide application of non-invasive continuous BP measurement models. However, a more diverse and bigger dataset and long-term monitoring experiments are needed to further test the generalization ability and robustness of the models.

## Figures and Tables

**Table 1:** Parameters of SBP and DBP models using support vector regression (SVR) and comparison between before and after GA optimization, the best mean squared error (Best MSE) represented the model accuracies.

	SBP Models				DBP Models			
	*c*	*γ*	*ε*	Best MSE	*c*	*γ*	*ε*	Best MSE
SVR (default)	1	0.07	0.1	337.37	1	0.07	0.1	40.83
GA-SVR	99.99	16.33	0.73	38.33	61.76	11.97	0.41	5.73

**Table 2:** Mean impact value (MIV) ranking of features regarding the SBP estimation model using genetic algorithm optimized support vector regression (GA-SVR).

Rank	Feature	MIV	Relative Contribution Ratio (%)	Cumulative Contribution Ratio (%)
1	*K*	2.8615	17.16	17.16
2	*C_slope_*	2.3776	14.26	31.42
3	*S_1_*	2.0928	12.55	43.97
4	HR	1.9194	11.51	55.49
5	*H_gr_*	1.8611	11.16	66.65
6	*S_2_*	1.6514	9.90	76.55
7	*H_er_*	1.4704	8.82	85.37
8	PTTc	0.7645	4.59	89.96
9	*S_1_/S_2_*	0.6640	3.98	93.94
10	*T_downr_*	0.3050	1.83	95.77
11	PTTb	0.2938	1.76	97.53
12	PTTa	0.2895	1.74	99.27
13	*H_ar_*	0.1065	0.64	99.90
14	*T_upr_*	0.0161	0.096	100

**Table 3:** MIV ranking of features regarding the DBP estimation model using GA-SVR.

Rank	Feature	MIV	Relative Contribution Ratio (%)	Cumulative Contribution Ratio (%)
1	HR	1.0485	15.07	15.07
2	*T_downr_*	0.8756	12.58	27.65
3	*H_gr_*	0.8706	12.51	40.16
4	*H_er_*	0.7255	10.43	50.59
5	*S_1_*	0.6119	8.79	59.38
6	*S_2_*	0.5415	7.78	67.17
7	*C_slope_*	0.5098	7.33	74.49
8	*K*	0.4878	7.01	81.50
9	PTTc	0.4217	6.06	87.56
10	PTTb	0.3577	5.14	92.70
11	*T_upr_*	0.2265	3.25	95.96
12	PTTa	0.1269	1.82	97.78
13	*S_1_/S_2_*	0.1009	1.45	99.23
14	*H_ar_*	0.0536	0.77	100

**Table 4:** Parameters of SBP and DBP models using GA optimized SVR and comparison between before and after MIV dimensionality reduction, the Best MSE represented the model accuracies.

	SBP Models				DBP Models			
	*c*	*γ*	*ε*	Best MSE	*c*	*γ*	*ε*	Best MSE
GA-SVR	99.99	16.33	0.73	38.33	61.76	11.97	0.41	5.73
GA-MIV-SVR	95.89	53.18	0.6486	30.98	98.33	14.26	0.99	4.68

**Table 5:** Performance comparison of the models. Abbreviations: mean absolute deviation (MAD); standard deviation (STD).

	SBP Error (mmHg)			DBP Error (mmHg)		
	MAD	STD	Error	MAD	STD	Error
GA-MIV-SVR	3.27	5.52	3.27 ± 5.52	1.16	1.97	1.16 ± 1.97
MLR	9.93	17.16	9.93 ± 17.16	2.59	4.38	2.59 ± 4.38
PTTc-SVR	13.13	21.73	13.13 ± 21.73	3.40	5.76	3.40 ± 5.76

**Table 6:** Grading of British Hypertension Society (BHS) system and the grades of different models based on the cumulative error.

	Model	Cumulative Error (%)			Grade
		≤5 mm Hg	≤10 mm Hg	≤15 mm Hg	
BHS	SBP/DBP	60	85	95	A
		50	75	90	B
		40	65	85	C
GA-MIV-SVR	SBP	77.8	96.7	99.3	A
	DBP	98.7	100	100	A
MLR	SBP	29.4	56.9	82.4	C
	DBP	92.2	100	100	A
PTTc-SVR	SBP	20.3	47.1	64.1	C
	DBP	79.1	96.1	98.0	A
